# Exploring issues in caregivers and parent communication of sexual and reproductive health matters with adolescents in Ebonyi state, Nigeria

**DOI:** 10.1186/s12889-019-8058-5

**Published:** 2020-01-17

**Authors:** Chinyere Ojiugo Mbachu, Ifunanya Clara Agu, Irene Eze, Chibuike Agu, Uche Ezenwaka, Nkoli Ezumah, Obinna Onwujekwe

**Affiliations:** 1 Health Policy Research Group, University of Nigeria Enugu, Enugu, Nigeria; 2 Department of Community Medicine, University of Nigeria Enugu Campus, Enugu, Nigeria; 3 Department of Health Administration and Management, University of Nigeria Enugu Campus, Enugu, Nigeria

**Keywords:** Parent- adolescent communication, Sexual and reproductive health, Adolescents

## Abstract

**Background:**

Parent-child communication is an effective tool for fostering healthy sexual and reproductive behaviours among adolescents. However, the topic is underexplored in Nigeria. This study examines how parents and caregivers communicate sexual and reproductive health-related matters with adolescents aged 13–18 years in Nigeria.

**Method:**

The study was undertaken in six communities in Ebonyi state, Nigeria using quantitative and qualitative research methods. Data were collected through, i) cluster randomized survey of 1057 adolescents aged 13–18 years, ii) twelve sex-disaggregated focus group discussions with adolescents aged 13 to 18 years, and iii) eight in-depth interviews with parents and caregivers. Univariate and bivariate analysis were performed for quantitative data, while qualitative data were analysed using thematic framework approach.

**Results:**

Less than half (47.9%) of adolescents in the survey reported ever discussing sex-related matters with anyone. Three-quarters of those who had this discussion did so with a friend/peer and this had significant correlation with sex/gender (*p* = 0.04). Out of 1057 adolescents who participated in the survey only 4.5% had ever discussed sex-related matters with a parent and this correlated significantly with wealth index (*p* = 0.003). Findings from qualitative interviews show that sex-related discussions between parents and adolescents are sporadic, mostly triggered by unpleasant occurrences, and consist of, i) information on pubertal changes, ii) warnings against intersex relationships and premarital sex, iii) promotion of abstinence, and iv) warnings against teenage pregnancy and unsafe abortion. Some parents were of the opinion that sex-related matters should not be discussed with adolescents because it could be interpreted as tolerance for sexual promiscuity. Overall, parents expressed that their capacity to discuss sex-related matters with adolescents is limited by lack of knowledge, and restrictive religious and cultural norms about adolescent sexuality.

**Conclusion:**

Communication between parents and adolescents on sexual health and reproductive-related matters rarely occurs. However, when it does, it mostly consists of strict warnings that may not protect adolescents from making unhealthy sexual and reproductive health choices. Interventions to improve parent-adolescent communication of sexual and reproductive health (SRH) should aim at improving parents’ capacity to communicate sexual and reproductive health matters, and deconstructing sociocultural norms around adolescent sexuality.

## Background

Effective communication is an essential aspect of adolescent sexual and reproductive health because it promotes good sexual and reproductive health choices and behaviours [1]. Adolescents aged 10 to 19 years engage in negative sexual practices which predispose them to various sexual and reproductive health risks [1] and considerable numbers of adolescents lack basic knowledge on sexual and reproductive health matters and approaches to avoid negative sexual practices [2]. Factors contributing to high rates of negative sexual practices among adolescents are diverse and have been attributed to family factors, social context, and poor communication [1]. Several studies support the assertion that the family is a primary source of information to adolescents on SRH matters [3, 4]. Family-initiated communication, especially parental, should be the principal channel for conveying knowledge and values to adolescents, including information related to their sexual and reproductive health (SRH).

The relationship between parents and their children is one of the most influential and meaningful relationships in the lives of adolescents [5]. Although parent-child relationships evolve over developmental periods of childhood and adolescence, parent-child communication strengthens bonds between parents and their children and it enables affection, builds self-esteem, mutual respect, and creates an environment of support for adolescents [5, 6]. Communication in the family setting is the ability to discuss and attend to the changing needs, feelings and desires of family members in a manner that is helpful and encouraging [7, 8]. The quality of parent-child communication significantly contributes to the quality of parent–child relationship and predicts subjective well-being of adolescents [9].

Parental discussion about adolescents’ sexual and reproductive health (SRH) is very important because adolescents who discuss sex-related matters with their parents are less likely to engage in negative sexual practices [10, 11]. Conversely, those who do not engage in discussions of sex-related matters with their parents are more likely to make poor sexual and reproductive health choices [10]. Adolescents have been found to rely mostly on peers and media as sources of information on sexual and reproductive health (SRH). Unfortunately, information obtained from these sources are either inadequate or false which is a major cause of poor adolescent SRH knowledge [2].

Effective parent-child communication is positively associated with reduction in risky sexual practices that are detrimental to adolescent health and well-being. However, this topic is underexplored in the Nigerian context. Titiloye and Ajuwon employed quantitative methods to estimate SRH discussion between adolescents and their parents [2], and this is limited in depth of information about triggers of parent-child communication, as well as societal and gender norms around discussing sex-related issues with adolescents. Although Izugbara employed qualitative methods to examine how and why these discussions happen, the study was conducted in rural areas alone limiting its transferability to non-rural settings [12].

This paper provides new knowledge on whether and how parents/caregivers communicate sexual and reproductive health-related matters with adolescents in rural and urban areas. It highlights the contents of this discussion, when it happens, including topics that are considered as taboos, and factors that trigger such communication. It also examines gendered perceptions of roles of parents in communicating sex-related matters with adolescents. This information could be useful for decision-makers in choosing strategies for improving parent-child communication of SRH matters. Implementation scientists may also find this paper useful for designing strategies to improve adolescents’ access to sexual and reproductive health information.

## Methods

### Study design and study area

This was a mixed-method study comprising of quantitative and qualitative methods. The study was undertaken in six communities selected from six local government areas (LGAs) in Ebonyi State, southeast Nigeria. The LGAs were purposively selected to ensure geographic and geopolitical spread in terms of place of residence (urban and rural) and senatorial zones. From each senatorial zone, we selected 2 LGAs that have been prioritized by Ebonyi State government for interventions in adolescent sexual and reproductive health. These LGAs were listed by stakeholders as having the highest rates of unwanted teenage pregnancies and abortions in the State. One community was selected from each LGAs.

Over 40% of Ebonyi state’s total population of about 2.8 million are under the age of 15 years. With an estimated annual growth rate of 2.7%, it is estimated that the population of adolescents will double by the year 2050 [13]. The 2013 NDHS report shows that maternal mortality rate among girls aged 15–19 is 30.5%; and 9.6% of girls in this age group have already begun child bearing [14].

### Study population, sampling and sample size

The study population consisted of unmarried adolescent boys and girls aged 13 to 18 years living in selected households, and parents/caregivers of these adolescents. Underage adolescents [13–17 years] whose parents/caregivers were not available to give consent at the time of the survey were excluded from being interviewed. The study also excluded household guests, and adolescents who had sight, mental or hearing impairments.

The respondents to the quantitative survey were selected using random sampling, whilst the respondents to the qualitative survey were selected using purposive sampling. In order to achieve a 5% precision at 95% confidence interval for population > 100,000 a minimum sample size of 400 was determined from Glenn’s table^1^ of sample sizes that would be necessary for given combinations of precision, confidence level and variability for different population sizes. This was doubled to enable sub-group (urban-rural) analysis of data, and further increased to 1100 for robustness and to account for incomplete responses or errors in questionnaires. Modified cluster sampling technique was used to select households from which eligible adolescents were recruited. One cluster (defined as an autonomous village^2^) was selected from each community. Within each cluster, the nearest public facility from the main entrance was identified as the starting point from which households were consecutively selected. All eligible adolescents in selected households were invited to participate in the survey.

Twelve FGDs with adolescents and eight IDIs with parents of adolescents were conducted. We purposefully selected older adolescents (15–18 years) who were not schooling and appeared to be well informed during the survey, then invited them to participate in focus group discussions. Other adolescents were randomly selected from government secondary schools in study communities. The FGDs were held with both in and out of school adolescents but disaggregated by sex, such that there were six FGDs each for boys and girls. Each sex-disaggregated focus group comprised of both in-school and out-of-school adolescents. Number of participants in FGDs ranged from 8 to 13. For the IDI with parents, we planned to interview two parents (one male and one female) of adolescents from each study community but ended up with eight parents due to refusals and delays in scheduling interview appointments within the study period.

### Research variables

The variables of interest in the quantitative study were: i) demographic characteristics such as place of residence, gender, schooling and wealth index; ii) discussion of sex-related matters – with parents, peers, etc.; iii) frequency of discussion of sex-related matters. Variables of interest for qualitative study include content of SRH communication, triggers, enablers and constraints, societal (gender) norms/nuances. The variables of interest were derived from studies that explored discussion of sexual and reproductive health matters between parents and their adolescents [10, 15].

### Data collection

Quantitative data: A structured questionnaire which was adapted from WHO illustrative questionnaire for interview-surveys with young people [16], was used to collect information from the adolescents. The questions focused on whom they have had sex-related communications and how often this happens. Fifty-four research assistants were recruited and trained for 5 days to assist with administering the questionnaire. Paper and electronic copies of the questionnaire were used to collect data over a period of ten days. Electronic copies of the questionnaires were uploaded to android tablets using SurveyCTO. Individual matching of information on completed paper-questionnaire with corresponding electronic-questionnaire was done before and after uploading data to the server and data was viewed concurrently.

Qualitative data: The interview guides for adolescent focus group discussion (FGD) and in-depth interview (IDI) with parents were developed by a team of qualitative research experts (see Additional files 1 and 2). Prior to commencement of the project in Ebonyi state, a relationship was established with some of the participants who attended a stakeholder engagement meeting held in the state. They were then reviewed by a team of content experts to ensure sufficient coverage of the subject area. The qualitative interviews were conducted by experienced qualitative researchers over a period of one month. Each interview was audio recorded, and hand-written notes were taken. All data collection instruments were pretested in a contiguous site among adolescents aged 13–18 years and their parents.

### Data analysis

Following data cleaning, 1045 questionnaires were judged to be completely filled and without errors, giving a response rate of 95%. Descriptive analysis was performed using Stata software and weighted proportions reported for categorical variables. Communication of sex-related matters were disaggregated by socio-demographic characteristics such as place of residence (urban or rural), gender, schooling, & wealth index to highlight distribution as well as to test for associations. Chi-square and *p*-values are reported for the multi-way table.

Household wealth index was calculated and this is a composite of household consumption pattern that places households on a continuous scale of relative wealth. Total household consumption was calculated by adding food and non-food expenditure. Per capita household consumption was then calculated by dividing total household consumption by number of people in the household. The per capita household consumption was used to classify households into socio-economic quintiles, Q1 to Q5, where Q1 refers to poorest households and Q5 refers to richest households.

**Fig. 1:**
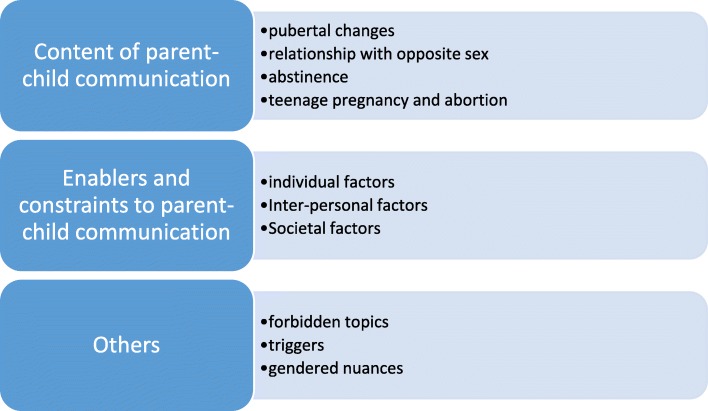
Coding scheme for parent child communication of sex-related matters

Audio files of qualitative interviews were transcribed verbatim in the language of the interview, then translated to English. All transcripts were processed and edited using Microsoft Word. More than seven unique codes were developed and used to anonymize each transcript. Data were analysed using a thematic framework approach (Fig. 1). At first, all the transcripts were read to get a general sense of the data. Then the richest FGD and IDI transcripts were selected for detailed study and coding. Key themes and sub-themes relating to parent-child communication of sexual and reproductive health matters were generated and this formed the initial coding framework. The framework was tested on two new transcripts (one of FGD and IDI) and refined into a final coding framework which was then applied to all the transcripts (including the four that were used to generate and test the framework). After analysis, the various findings were presented to key stakeholders in Ebonyi state through a workshop for validation of synthesized data.

## Results

Table 1 highlights the background information of surveyed adolescents. Urban-rural disaggregation shows that 551 (50.7%) people were from urban areas while 494 (49.3%) were from rural areas. There were 598 girls (57.2%) and 447 boys (42.8%). Majority of them 966 (92.4%) were attending school at the time of the survey. With respect to employment, out of 502 adolescents who reported they had ever worked, 262 (52.5%) were currently employed. This essentially translates to one-quarter of adolescents in the survey.

**Table 1:** Demographic characteristics of surveyed respondents

Variables (*N* = 1045)	Frequency (n)	Weighted percent (%)
Place of residence		
Urban	551	50.7
Rural	494	49.3
Gender		
Female	598	57.2
Male	447	42.8
Schooling		
In-school	966	92.4
Out-of-school	79	7.6
Wealth index		
Q1	224	21.9
Q2	211	20.6
Q3	214	20.0
Q4	198	18.8
Q5	197	18.6

N = denominator (number of people that responded to the question); n = numerator/frequency (number of observations for each outcome)

Table 2 shows that less than half of the adolescents, 500 (47.9%), reported ever discussing sex-related matters with anyone. Majority of those who have had this discussion, 388 (77.7%), did so with friends. Three hundred and forty of them (68.1%) reported that the discussions happened occasionally.

**Table 2:** Communication of SRH issues among surveyed respondents

Variables	N	Frequency (n)	Weighted percent (%)
	1045		
Ever discussed sex-related matters with anyone		500	47.9
Never discussed sex-related matters with anyone		545	52.1
*Person with whom sex was discussed	500		
Friends		388	77.7
Teacher		78	15.3
Siblings		50	10.0
Mother		47	9.4
Other family members		42	7.4
Health workers		7	1.4
Other people		36	0.7
Frequency of discussion of sex-related matters	500		
Weekly		76	15.0
Monthly		60	12.1
Yearly		10	1.9
Occasionally (when something happens)		340	68.1
Others		13	2.7
Refused to say		1	0.1

^*^Multiple response allowed

Results of bivariate analysis of demographic factors associated with communication of sex-related matters between adolescents and their parents (mother) are shown in Table 3. Statistical significant associations were found, i) when ‘ever discussed sex-related matters’ was cross-tabulated with adolescents’ schooling status (*p* = 0.03); ii) ‘when discussed sex-related matters with mother’ was cross-tabulated with wealth index (*p* = 0.003); and iii) when ‘discussed sex-related matters was cross-tabulated with gender (*p* = 0.04).

**Table 3:** Relationship between demographic characteristics of adolescents and communication of sex-related matters

Demographic characteristics	Ever discussed sex-related matters with anyone		Discussed sex-related matters with parent (mother)		Discussed sex-related matters with friends or peers	
	N	n (%)	N	n (%)	N	n (%)
Residence						
Urban	551	255 (46.2)	255	28 (11.0)	255	200 (78.7)
Rural	494	245 (49.6)	245	19 (7.8)	245	188 (76.7)
χ^2^ (*p* value	1.18 (0.28)		1.58 (0.21)		0.27 (0.59)	
Gender						
Female	598	296 (49.5)	296	34 (11.5)	296	221 (74.6)
Male	447	204 (45.8)	204	13 (6.2)	204	167 (82.2)
χ^2^ (p value	1.41 (0.24)		3.93 (0.05)		4.03 (0.04)*	
Schooling						
In-school	966	453 (46.9)	453	41 (9)	453	348 (76.9)
Out-of-school	79	47 (59.3)	47	6 (12.8)	47	40 (85.2)
χ^2^ (p value	4.55 (0.03)*		0.73 (0.40)		1.72 (0.19)	
Wealth index						
Q1	224	107 (47.7)	107	47 (4.6)	107	82 (76.4)
Q2	211	106 (50.4)	106	3 (2.9)	106	80 (75.4)
Q3	214	109 (50.8)	109	12 (11.1)	109	83 (76.4)
Q4	198	96 (48.6)	96	16 (16.7)	96	77 (80.2)
Q5	197	81 (41.2)	81	11 (13.6)	81	65 (80.7)
χ^2^ (p value	4.78 (0.31)		16.38 (0.003)*		1.28 (0.86)	

^*^p-value < 0.05

### Contents of parent-child communication about sexual and reproductive

Parents and adolescents were asked to describe the topics that are discussed when they have conversations on SRH matters. The responses from parents and adolescents were similar, and they identified that common topics discussed include, pubertal changes, relationship with opposite sex, abstinence from premarital sex, and teenage pregnancy and abortion.

#### Pubertal changes

With respect to discussions on pubertal changes, some parents reported that they tell their adolescents about changes they can expect to see in their bodies as they transform from children to adults.

*“...when you know that your child, both male and female is up to 13 years and above, we will educate them as they are getting to adult* [about the] *physical changes and development that will come. For example as a girl, you know between the ages of 12, 13, 14, 15 she will start seeing her menstruation”* (Female parent - PAAB)

#### Relationships with opposite sex

Parents reported that they also warn their adolescents to avoid relationships with the opposite sex because it would ultimately result to unwanted pregnancy and early marriage. Some adolescents re-echoed this, stating that they are often warned by their parents not to mingle with people (including age or school mates) of the opposite sex. For instance, adolescent girls are warned to avoid boys or risk becoming pregnant and getting married off to someone they may not like to marry, or getting an abortion and losing their lives. Boys, on the other hand, are warned not to date until they have completed their education and are capable of taking care of a family. Supporting quotes from a parent and two adolescents include,

*"We advise them to stay away from men and boys. That the implication of that thing is pregnancy which if they abort may cost them their lives and if they keep it might warrant marrying someone they do not like to marry. So you have to avoid it* (sexual intercourse) *until you are through with your school, so that you can get somebody you like“*(Female Parent - PAIK)

*"*[we are told] *not to date a girl when we cannot take care of the girl or a family* [and that if we do] *it can lead to school dropout"* (Male Adolescent - ADAFM)

*“My mother advises me against relationship with the opposite sex and against abortion services if I should get pregnant"* (Female Adolescent - ADABF)

#### Abstinence from premarital sex

Adolescents mentioned that their parents and caregivers tell them to abstain from premarital sex because it leads to sexually transmitted infections and unwanted pregnancy which results in school drop-out. Adolescents also highlighted that that their parents do not tell them about other forms of contraception, rather they are told that it is morally wrong to engage in premarital sex; and girls are specifically advised to dress modestly and be mindful not to engage in other behaviours that may attract the attention of the opposite sex (boys). Supporting quotes from adolescents include,

*"They* (parents) *don’t tell us about condoms, rather on how to abstain from it (sexual intercourse) because of sexually transmitted disease and unwanted pregnancy … ”* (Female Adolescent - ADAFF)

*"...Mother always advises me to stay away from sex. Her reason is so that I do not get a girl pregnant at this my early age; because it can lead to school dropout … ”* (Male Adolescent - ADAFM)

*“My mother tells me to avoid boys and premarital sex because any interaction with a boy is a sin”* (Female Adolescent - ADOHF)

*"My Aunty* [guardian] *advises me to be cautious of the dresses I put on while going out to avoid falling into the hands of boys who are bad* [and can get me pregnant] *since this will bring disgrace to the family"* (Female Adolescent - ADABF)

#### Teenage pregnancy and abortions

Parents and adolescents reported that immediate and long term effects of unwanted teenage pregnancy is a topic that features very commonly in their discussions. Parents often remind their adolescents that teenage pregnancy could result in abortion, school drop-out, and sexually transmitted infections, all of which have negative implications for their prospects in life. Adolescents also reported that their parents tell them that they are unable to care for themselves, let alone a baby.

They are also warned about life threatening consequences of unsafe abortions, and were advised to avoid it at all cost.

*" …* [My mother] *warns me about unwanted pregnancy, that I should avoid it because a girl like me cannot take care of herself, talk less of a baby..."* (Female Adolescent, ADAFF)

In addition to the aforementioned topics that are commonly discussed between parents/caregivers and adolescents, peer influence and companionship are also discussed. Parents warn their children to avoid bad friends/companions so they do not become victims of negative peer influence.

### Forbidden topics on sexual and reproductive health

Parents were asked if there are sex-related topics that should never be discussed with adolescents. A few parents were of opinion that there are no forbidden topics. They felt that adolescents need to understand sexual and reproductive health issues and implications of making wrong decisions. This information, they believe, should be provided to adolescents at home so they are not misinformed by their friends. Some parents were of the opinion that matters around sexual intercourse should not be discussed with adolescents.

### Triggers of parent-child communication on sex-related matters

Adolescents were asked to describe how often they communicate with their parents on sex-related matters. They reported that the discussion only happens occasionally and is often triggered by unpleasant or tragic occurrences in the community, such as when a teenager gets pregnant or dies from an unsafe abortion. The discussions were also reported to start once parents notice that their adolescents are unusually mingling with friends of the same or opposite sex. Here are some quotes from male and female adolescents,

*“If my mother hears an unpleasant story outside, she will then come home and caution you”* (Female Adolescent - ADOHF)

*“Discussion with mum starts when she sees me around with a girl; that is the time she will like to give advice”* (Male Adolescent - ADAFM)

*"*[the discussion starts] *When something bad* [such as unwanted pregnancy] *happens* [to another young person in the community] *or maybe when you start going out to visit friends*" (Female Adolescent - ADOHF)

This finding from focus group discussions with adolescents was similar with the quantitative result where most (68.1%) adolescents reported occasional discussion on sex-related matters with their parents. However, parents were not asked to describe the frequency of discussion on sex related matters or what triggers SRH discussion with adolescents.

### Gendered nuances about parent-child communication of sexuality, sex-related matters and reproductive health

Some variations in the content of discussion between parents and their male and female adolescents were highlighted during FGDs with adolescents. Parent-child discussions around pubertal changes, abstinence, pregnancy and abortions resonated more during FGDs with girls; whereas discussions between parents and their adolescent boys included information on life skills, relationship building, making responsible decisions about sex, and using contraceptives. Some supporting quotes are,

*“They do not tell us about condoms but how to abstain from* (sex) *because of sexually transmitted diseases and unwanted pregnancy. She* (mother) *tells me the danger of engaging in premarital sex”* (Female Adolescent – ADAFF)

*“Some people usually have discussion on how they will manage themselves* (life skills)*. They* (parents) *would like to tell you more about pregnancy and contraceptives which you can use to avoid pregnancy”* (Male Adolescent – ADEZM)

*“*(We are told) *not to date if we cannot take care of a girl or a family because it could lead to dropping out of school”* (Male Adolescent – ADAFM)

Exploration of pairing patterns for parent-child communication of SRH matters revealed that there are gendered notions about parent-child pairing for sex-related discussions. Although most mothers expressed that they are able to communicate effectively with their adolescent boys and girls, they were of the opinion that fathers should discuss with their sons while mothers discuss with their daughters. This, in their view, would ensure uninhibited discussions which are usually more effective. Also, mothers are better positioned to understand their daughters, just like fathers their sons.

*“It is the responsibility of both father and mother but father will understand his son better than the mother likewise mother”* (Female Parent - PAIZ)

Although most respondents asserted that both parents have a role to play in adolescent sexuality education, there was a general perception that mothers are primarily responsible. One of the fathers categorically stated it is a woman’s responsibility to provide sexuality education to her sons and daughters. His reason is that fathers are usually unavailable to have this discussion because they are busy making money to provide for their families’ needs. Hence, mothers should take care of the children at home, monitor their behaviour, nurture and communicate with them at a young age. This finding is validated by the quantitative survey result as well as focus group discussions with adolescents where it was clearly stated that SRH/sex-related discussions were held with mothers rather than fathers.

*“It is the duty of both parents, but the mother has a very vital role to play. Because I as a man, there are certain thing I will not discuss with my daughter, but the mother will take time to educate the children”* (Male Parent - PAEZ)

*“Parents especially fathers find it difficult to mingle with this adolescents”* (Male Parent - PAIK)

### Challenges to parent-child communication about sexual and reproductive health

Parents expounded numerous challenges they encounter while discussing sexual and reproductive health matters with their adolescents and they include, i) individual factors such as personal inhibitions and lack of knowledge about what to communicate and how to communicate; ii) interpersonal factors such as limited opportunities to interact with adolescents, and generation gap; iii) restrictive cultural and religious beliefs.

Some parents reported that they do not know what to discuss (and the limits) with their adolescents when it comes to sex-related matters. Some also stated that they do not feel comfortable having casual discussions about sex with their adolescents. With respect to interpersonal factors, it was stated that parents do not spend quality time with their adolescents because they are busy. They were of the opinion that adolescents do not pay attention or want to discuss with their parents because of the generation gap. Religious and cultural norms restrict parents from having sex-related discussions with their children. From the findings some parents explained that talking about sex with adolescents is frowned upon by the church and community. A parent categorically mentioned that parents have to conform to traditions and norms of the community in which they are born, even when it comes to child rearing. Some quotes include,

*“As a father, when discussing with my child she talks less; nothing more than a ‘yes’ or ‘no’....”* (Male Parent - PAB)

*“Our church said we should not talk about sex because it affects our religion... In tradition, there is what is called norm and when you are born into a certain community you are influenced by the tradition of the community”* (Male Parent - PAB)

Adolescents also reported that they do not feel comfortable discussing their sexual lives with their parents because their parents are not open-minded or accepting of their view points, and they are easily misunderstood. On the other hand, parents reported that adolescents barely listen to their advice because they do not want to receive directions from their parents.

## Discussion

Our study highlights gaps in parent-child communication of sexual and reproductive health matters. We found that majority of adolescents had never discussed sex-related matters with their parents/caregivers and this is consistent with reports from similar studies [11, 17]. However, a few adolescents reported they have had SRH discussions with their mothers; though not statistically significant, more girls reported having had this discussion with mothers, compared to boys. Studies on parent-child communication have reported mothers as the usual (sometimes only) source of parental communication about SRH matters [4, 18]. In-depth exploration of the contents and triggers of parent-child communication revealed that discussions often centred on pubertal changes, relationship with opposite sex, abstinence from premarital sex, and teenage pregnancy and abortion, and were triggered by environmental factors such as unpleasant or tragic occurrences in the community and parental observations of budding relationships between adolescents of opposite sex. The content and triggers of sexual and reproductive health (SRH) discussions between parents and their children in this study also corroborate findings from similar studies [11, 17]. The scope of discussion between parents and their adolescents was found to be limited and this could explain why adolescents would prefer to discuss with their peers and friends who are ‘open-minded’, rather than their parents.

Discussions between adolescents and their parents in this study appeared to consist mostly of warnings about heterosexual relationship and its consequences on the health and well-being of adolescent girls. Some studies have reported that most discussions engaged by parents with their adolescents are precautionary and authoritative [17, 19]. These types of communication are ineffective in producing positive behaviors among growing ‘adults’. Similar to our finding of perfunctory discussions between parents and their adolescents, other authors reported that SRH discussions between parents and adolescents rarely occur, and that parents used unpleasant occurrences as opportunities to discuss with their adolescents [17, 20]. In most African cultures, including Nigeria, open discussions on sexual and reproductive health particularly sex-related matters are very scripted and deeply shrouded in mystery [4]. There is high intolerance for sexual promiscuity (and this includes pre-marital sex). Hence, parents communicate with their adolescents through warnings, threats and physical discipline, often times triggered by what they recently saw or heard from the community/environment [2]. The sacredness attached to discussions around SRH matters hinders parents from having meaningful relationships with their adolescents.

Whilst warning their children to abstain from premarital sexual affairs, parents in this study failed to discuss other contraceptive options with their adolescents. This corroborates studies that show that contraception is the least discussed topic between parents and adolescents [1, 12, 17]. Although some parents may consider some topics ‘forbidden’, the need to provide complete and comprehensive sexual and reproductive health information to adolescents cannot be underscored. Some parents in this study described the need to provide all sexual and reproductive health information to adolescents particularly at home to avoid misinformation and wrong choice of action. Evidence shows that adolescents who are exposed to correct and complete SRH information are more inclined to make good choices, and less vulnerable to the consequences of ignorance. Pluhar & Kuriloff in their study of African American mothers and their daughters reported that mothers who discussed contraceptive options (including condoms) with their daughters where able to avert unwanted teenage pregnancy or STIs [21].

Although parents in this study acknowledged that effective SRH discussion with adolescents could be accomplished by either a father or mother, notions about pairing patterns for parent-child communication was gendered. Studies in Africa reveal that parent-child communication of SRH is commonly done along gender lines [10, 22]. Hence, suggestions in the study were that pairing for such conversations should be sex-based, that is father-son and mother-daughter and that it is the woman’s role and responsibility to communicate SRH issues with her children (boys and girls alike). The reason for the latter is that she is the primary caregiver, nurturer and home maker. Baku et al., reported similar diversity in parents’ views of pairing for SRH discussions with adolescents. Most mothers preferred to have this discussion with their daughters because they are considered more vulnerable, while a few reported they would be more comfortable discussing with their sons [17, 20].

Challenges encountered in having SRH discussions between parents and adolescents ranged from individual to interpersonal and societal factors. Inability to communicate with adolescents on SRH has been reported among parents in some African studies and this is rooted in restrictive cultural and religious beliefs [2, 10, 17]. Parents experience inhibitions discussing sex-related matters with their children because they feel embarrassed to do so, lack courage and are concerned that it could be misinterpreted as tolerance for premarital sex [10, 20]. Parents also experience difficulty initiating SRH discussions with adolescents because they lack knowledge of what to say, and the necessary skills to communicate effectively [20, 23]. These challenges create potential entry points for interventions to improve parent-child communication of SRH matters. Future studies could examine the effectiveness of such interventions and potentials for scalability in similar settings.

Our study combined quantitative and qualitative methods of data collection which enabled in-depth exploration of parent-child communication. It reports data that was collected from both parents and adolescents and this enabled us tell the story from both sides of the isle. Furthermore, it exposes the gendered nature of parent-child communication (which needs further exploration), and highlights potential entry points for designing interventions. The strength of our findings could have been improved by a more robust quantitative component. However, we report important findings that could contribute to designing a more comprehensive structured questionnaire.

## Conclusion

Although parents in this study recognize the importance of providing information on sexual and reproductive health to adolescents, parent-child communication of sexual and reproductive health matters rarely occurs. The few adolescents who had this discussion did so with their mothers; this was often reactive and triggered by environmental factors. Sexual and reproductive health information provided by parents to their adolescents consist of strict warnings and ambiguous information, which are unlikely to instil confidence or protect adolescents from making poor health choices.

There is a need to reorient parents to communicate better with their adolescents, and initiate discussions on adolescent sexual and reproductive health. Knowledge building interventions will equip parents with the confidence they need to initiate this discussion as early as possible, and to adapt and sustain it at various stages of their adolescent’s development.

## Supplementary information

**Additional file 1.** FGD guide for adolescents.

**Additional file 2.** IDI guide for parents and guardians of adolescents.

## Data Availability

The study dataset is available on request.

## Abbreviations

ASRH: Adolescent Sexual and Reproductive Health
FGD: Focus group discussions
HPRG: Health Policy Research Group
IDI: In-depth Interview
IDRC: International Development Research Centre
SRH: Sexual and Reproductive Health

## Footnotes

1 Glenn D (1992). Sampling the Evidence of Extension Program Impact. Program Evaluation and Organizational Development, IFAS, University of Florida.

2 An autonomous village is defined as a community which is governed by an appointed or elected traditional ruler.
