# Supplementary material for: Exploring issues in caregivers and parent communication of sexual and reproductive health matters with adolescents in Ebonyi state, Nigeria
Source: BMC Public Health. 2020 Jan 17;20:77. doi: 10.1186/s12889-019-8058-5 (PMC6969441; doi:10.1186/s12889-019-8058-5)
Supplement: Supplementary file 1 — Additional file 1. FGD guide for adolescents. [file 12889_2019_8058_MOESM1_ESM.docx]

## Additional file 1: FGD guide for adolescents (male and female groups)

### Introduction, purpose and procedure

I am a trained data collector from Health Policy Research Group University of Nigeria Enugu Campus and we are working with Ebonyi State government to conduct a study on Adolescent Sexual and Reproductive Health.

We plan to be more involved with your community and engage with young people within the age range of 13 to 18 years over the coming months. We will like to talk with you about your sexual and reproductive health, and ability to access sexual reproductive health information and services.

Your participation in this discussion is important as it will help us achieve the aim of the study. All information given will be confidential, during and after the research process. Your participation is voluntary and you do not have to answer questions you do not wish to. If you have any question please ask them now or later at the end of the interview.

With your permission, I would like to record this interview to make sure I accurately capture our discussion. This interview will last about 45 minutes.

**Ground rules** (to be set by moderator and discussants)

### Discussion

**Sources of information on sexual and reproductive health**

1. Can you tell me about adolescents’ sources of information on sex, pregnancy and prevention of pregnancy? What are these sources of information?

***(Prompt for:*** *school teacher, mother, father, brother, sister, other family members, friends, Doctors, other health professionals, books/magazines, films/videos, social media, internet, mobile phone SMS etc)*

1. Which are their preferred sources of information about sex, pregnancy, and prevention of pregnancy? What are their reasons for preferring these sources?
   1. Which of these is the most important source of information? How is this so?
2. What types of discussion do adolescents have with parents (father/mother) or guardians about sex? What about you?
   1. What types of information are provided? What topics are discussed or not discussed?
   2. How frequent do these discussions hold?
   3. What triggers this discussion between you and your parents?
   4. Which of your parents (father or mother) do you usually have this discussion with? Why is this so?
   5. If you do not discuss SRH matters with your parents, what are your reasons?
3. What is the nature/type of sex education provided in school?

Probe

- What specific information is provided?
- Which group/level of students receive information in sex education

**Sexual and Reproductive Health Services**

1. Can you tell me about where adolescents go to receive the following services:

- Treatment of STIs;
- Contraceptive counselling and commodities
- Abortion or termination of unwanted pregnancy

**Probes**

- What types of health facilities do they visit? What are their reasons for using these types of facilities? *(****Prompts:*** *information delivery; able to ask questions; questions were answered adequately; privacy; cost; etc)*
- What types of health workers do they see?
