# Supplementary material for: Exploring issues in caregivers and parent communication of sexual and reproductive health matters with adolescents in Ebonyi state, Nigeria
Source: BMC Public Health. 2020 Jan 17;20:77. doi: 10.1186/s12889-019-8058-5 (PMC6969441; doi:10.1186/s12889-019-8058-5)
Supplement: Supplementary file 2 — Additional file 2. IDI guide for parents and guardians of adolescents. [file 12889_2019_8058_MOESM2_ESM.docx]

## Additional file 2: In-depth interview guide for parents and guardians of adolescents

### Introduction, purpose and procedure

I am a trained data collector from Health Policy Research Group University of Nigeria Enugu Campus and we are working with Ebonyi State government to conduct a study on Adolescent Sexual and Reproductive Health. We plan to be more involved with your community and engage with young people within the age range of 13 to 18 years over the coming months.

The aim of this interview is to get your views on parental involvement in provision of SRH information and advice to adolescents in Ebonyi state. Adolescent sexual and reproductive health refers to all matters relating to their safe sex life and capability to have children. The information you provide in this interview will be treated as confidential and your participation will be anonymized.

With your permission, I would like to record this interview to make sure I accurately capture our discussion. This interview will last about 45 minutes.

### Background characteristics

Interview code

Date of interview

Sex

Time start

Time stop

### Discussion

**Parental involvement in provision of SRH information and advice**

1. What is your view about adolescent sexual and reproductive health needs?

*(****Prompts:*** *prevention of unwanted pregnancy, abortion, STIs and HIV/AIDS, access to accurate and appropriate information, counselling, forming relationships, contraceptives, etc)*

**Probes:**

- Why do you hold these views?
- Do you think adolescents’ have unique/special SRH needs?
- Do the SRH needs of boys differ from those of girls? How is this so?

1. Can you tell me about where your adolescents get their SRH information from?

*(****Prompts****: friends, counsellors, media, church, teachers, family members, internet, mobile phone SMS etc.)*

**Probes:**

- What do you think about these sources of information in terms of adequacy and appropriateness of information shared/provided?
- How important are these sources of information (that you have mentioned) to adolescents?

1. Can you tell me about who and what you think has the **most** influence on your adolescent’s SRH?

***(Prompts:*** *Peer influence, parental/family member influence, media influence, religious influence, internet influence, celebrity/artists’ influence, popular culture, etc)*

**Probes:**

- In what way(s) does this person or source influence your adolescents?
- How important is this person or source of influence on your adolescents SRH?

1. What kinds of discussions do you have with your adolescents about SRH matters?

*(****Prompts:*** *physiological and anatomical changes during puberty; boyfriend/girlfriend relationships; sexuality – orientation, exposure, use of protection (also issues about contraception and contraceptives), assertiveness; peer group influence, etc)*

**Probes:**

- Are there some topics on SRH matters that you think should not be discussed with adolescents? What are these topics and why is this so?
- What do you tell them and what do they tell you
- What challenges do you face in discussing SRH matters with your adolescents? *(Prompt for religious bias)*
- Are you more comfortable discussing with your daughter or sons or wards?
- Whose responsibility is it in the home (father or mother or other relative) to discuss SRH matters with adolescents? Why is this so?
- If you do not discuss SRH matters with them, what are your reasons?

**Perception of sexual and reproductive health services**

1. What is your view on the importance of providing SRH services for adolescents?

**Probes:**

- What services are available and where?
- Are the services adequate and appropriately delivered?
- Are the health workers trained/skilled to deliver SRH services to adolescents?
- Are the health facilities equipped to meet the SRH needs of adolescents (including need for privacy)?
